# Heterologous Expression of a *Neurospora crassa* Catalase Reprograms the Cellulase System and Enhances β-Glucosidase Production in *Trichoderma reesei*

**DOI:** 10.3390/jof12080554

**Published:** 2026-07-25

**Authors:** Haowen Sun, Changbin Tang, Yifan Chen, Yao Zhang, Wenjin You, Shufen Su, Hanfei Zhao, Jianzhong Huang, Xianzhang Jiang, Lina Qin

**Affiliations:** National and Local Joint Engineering Research Center of Industrial Microbiology and Fermentation Technology, College of Life Sciences, Fujian Normal University, Fuzhou 350108, China; sunhaowen96@163.com (H.S.); tcb13705078586@outlook.com (C.T.); yifanchen0107@163.com (Y.C.); yaozhang1231@163.com (Y.Z.); youwenjin657@163.com (W.Y.); 18859709047@163.com (S.S.); 18558959177@163.com (H.Z.); hjz@fjnu.edu.cn (J.H.)

**Keywords:** *Trichoderma reesei*, *Neurospora crassa*, catalase, cellulase production, β-glucosidase

## Abstract

Efficient saccharification of lignocellulose, the most abundant renewable carbon reservoir resource, is of great industrial importance. *Trichoderma reesei* is a premier cellulase producer, but its fermentation efficiency is often constrained by dual challenges: dissolved oxygen limitation and intrinsic oxidative stress. To address this, we engineered *T. reesei* to heterologously express a robust catalase gene (*cat-3*) from *Neurospora crassa*. The recombinant strain *Tr-cNcat3* exhibited a 7.4-fold increase in extracellular catalase activity. *Tr-cNcat3* showed an increase in total extracellular protein, resulting in markedly enhanced filter paper activity (FPA) and β-glucosidase activity compared to the control. Strikingly, this intervention specifically triggered a significantly higher expression of β-glucosidase, a known bottleneck in *T. reesei*’s cellulase system, particularly on bagasse and straw as the carbon source. Moreover, the ability of the supernatant to degrade cellulose substrates was improved. Our results reveal that overexpression of *cat-3* in *T. reesei* could modify the cellulase cocktail by triggering a higher level of β-glucosidase. This study provides a novel and effective genetic engineering strategy to unlock the full industrial potential of *T. reesei* for cost-effective lignocellulosic biorefining.

## 1. Introduction

*T. reesei* is a premier source of industrial cellulases [1]. Its secreted enzyme system—comprising cellobiohydrolases (CBHs), endoglucanases (EGs), and β-glucosidases (BGs)—functions synergistically to convert cellulose into fermentable glucose, a key feedstock for biofuels [2,3,4]. During large-scale fermentation, the high consumption of oxygen leads to oxidative stress, which significantly impairs fungal growth and enzyme activity. Therefore, optimizing oxygen supply remains a pivotal challenge in *T. reesei*’s industrial potential. Conventional solutions, such as increasing oxygen concentration and raising the agitation speed of fermenters [5,6], have been employed. While these methods can partially alleviate the issue of insufficient oxygen supply during fermentation, they simultaneously introduce problems of high cost and excessive peroxide accumulation [7,8].

Reactive oxygen species (ROS), including superoxide anion (O_2_^−^), hydrogen peroxide (H_2_O_2_), and hydroxyl radical (•OH), are generated through cellular metabolic processes such as the mitochondrial electron transport chain and NADPH oxidases [9,10]. Excessive ROS can directly attack macromolecules within the cell—including proteins, lipids, and DNA—leading to the disruption of cellular structures and functions [11,12,13]. For instance, H_2_O_2_ can induce lipid peroxidation, compromising membrane integrity and resulting in the leakage of cellular contents and cell death. Furthermore, H_2_O_2_ inhibits the activity of key enzymes, such as those involved in energy metabolism and protein synthesis, thereby restraining fungal growth [14,15,16]. Specifically, H_2_O_2_ can oxidize the structural domains or active sites of cellulases, causing their inactivation [16,17]. Moreover, H_2_O_2_ disrupts cellular energy metabolism and amino acid synthesis, thereby reducing the precursors and energy supply necessary for cellulase production [18]. Cells possess defense mechanisms involving antioxidant enzymes (e.g., superoxide dismutase, catalase, and glutathione peroxidase) and small molecule antioxidants (e.g., glutathione and vitamin C) to scavenge H_2_O_2_ [19,20]. However, under conditions of oxidative stress, these antioxidant systems can be overwhelmed by the excessive load of H_2_O_2_, leading to a further exacerbation of oxidative damage [21,22].

Catalase (CAT) is one of the crucial antioxidant enzymes in living organisms, catalyzing the decomposition of H_2_O_2_ into water and oxygen, thereby protecting cells from oxidative damage [23]. In filamentous fungi, Catalase-3 (*cat-3*), as a significant member of the catalase family, plays an important role in countering oxidative stress, regulating metabolism, and enhancing environmental adaptability. Studies have shown that *cat-3* activity is closely associated with fungal growth, development, and responses to external stressors [24,25]. For example, upon exposure to oxidative stress inducers (such as hydrogen peroxide or hydroquinone), the expression of *cat-3* is significantly upregulated to bolster the cell’s antioxidant capacity [26]. Therefore, we hypothesized that elevating *cat-3* expression in *T. reesei* would enhance its antioxidant capacity and consequently reduce peroxide accumulation in the growth environment. Based on the above assumptions, this study used genetic engineering to heterologously express *cat-3* from *N. crassa* in *T. reesei*. The selection of *N. crassa cat-3* stems from its superior performance over other fungal catalases. While *N. crassa* encodes multiple catalases (*cat-1*, *cat-2*, *cat-3*, *cct-1*/*cat-4*) [27,28,29,30], *cat-3* is indispensable for mycelial growth and uniquely responsive to diverse oxidative stresses [31,32].

This study demonstrates that heterologous expression of *N. crassa’s cat-3* gene in *T. reesei* not only improves the culture environment and enhances cellulase production through its antioxidant properties, but also unexpectedly optimizes the cellulase system by stimulating the production of β-glucosidase, thereby increasing *T. reesei’s* industrial potential for biofuel and bio-based chemical production. Additionally, our findings could provide theoretical basis for understanding the association between redox regulation and the expression of cellulase systems.

## 2. Materials and Methods

### 2.1. Microbial Strains and Growth Conditions

Escherichia coli *DH5α* was used for plasmid construction. *T. reesei Tu6Δku70* (a gift from Dr. Monika Schmoll) was the host. The *Cpyr4* strain was constructed by transforming *Tu6Δku70* with *pSK-pyr4* [33]. The *Tr-cNcat3* strain was generated by integrating the *cat-3* ORF from *N. crassa* (under *PcDNA1* and *Tcbh2*) into the *cel3c* locus of *T. reesei*.

Strains were grown on PDA (with or without 10 mM uridine) at 28 °C for 5–6 days. Minimal medium (MM) [34] was used for selection. For protein expression, conidia (10^6^/mL) were inoculated into 100 mL MM + 2% glucose, shaken at 220 rpm, 28 °C for 48 h. Mycelia were filtered, washed, and ~2.3 g wet weight transferred to 50 mL MM containing 2% carbon source (bagasse, Avicel, straw, or lactose), then incubated at 28 °C, 220 rpm.

### 2.2. Transformation of T. reesei Strains by Electroporation

Transformation was performed by electroporation as described [35]. Briefly, fresh conidia (within 7 days) were washed with ice-cold 1.1 M sorbitol, resuspended to 10^8^ conidia/mL. A total of 1–2 μg DNA was mixed with 90 μL conidia suspension in a pre-chilled cuvette. Electroporation used Gene Pulser Xcell (Bio-Rad Hercules, CA, USA) at 1.6 kV, 600 Ω, 25 μF. After pulsing, 900 μL ice-cold sorbitol was added, then transferred to 9 mL YPD and incubated 12 h at 30 °C. Cells were centrifuged, resuspended in 500 μL YPD, mixed with 14 mL top agar, and spread on MM plates. Colonies appeared after 5–7 days at 30 °C.

### 2.3. Determination of Biomass in Culture Including Insoluble Cellulose

The linearized knockout cassette, containing the *N. crassa cat-3* ORF expression module (flanked by PcDNA1 promoter and Tcbh2 terminator) and the *pyr4* selection marker, was flanked by the ~1.5 kb upstream and downstream homology arms of the *cel3c* locus to direct site-specific integration via double-crossover homologous recombination.

### 2.4. Construction and Verification of Recombinant Strains

Biomass from insoluble cellulose cultures was estimated by intracellular protein. Mycelia were harvested by vacuum filtration, and bead-beaten in 0.1 M NaOH for 1 min at room temperature. After centrifugation, protein in supernatant was measured by Bradford assay (Bio-Rad).

### 2.5. SDS-PAGE Analysis and Protein Concentration Assays

SDS-PAGE was performed with One-Step PAGE Gel Fast Preparation Kit (Shanghai Meiji Biotechnology Co., Ltd., Shanghai, China). Total protein in supernatants was quantified by Bradford method (Bio-Rad).

### 2.6. Enzyme Activity Assays

Filter paper activity (FPase) and β-glucosidase activity (pNPGase) were measured according to IUPAC standard methods [36]. FPase: 40 mg Whatman No.1 filter paper in 0.05 M citrate buffer (pH 4.8) at 50 °C for 60 min, reducing sugars quantified by DNS. β-glucosidase: 50 mM pNPG in same buffer at 50 °C for 30 min, pNP measured at 405 nm. One unit = 1 μmol product per min.

### 2.7. Enzymatic Saccharification of Biomass

Substrates: Sugarcane bagasse, corn straw, rice straw, and Miscanthus were obtained from Guangxi Huabao Fiber Products Co., Ltd. (Laibin, China). Materials were washed, air-dried, milled, and sieved to obtain particles between 40 and 60 mesh. No chemical pretreatment was performed. Xylan (from beechwood) and Whatman No.1 filter paper were used as model substrates.

Hydrolysis used crude enzymes from 7-day cultures. The supernatant was filtered through a 0.22 μm membrane. For each substrate, the reaction mixture (30 mL) contained: 0.6 g dry biomass (2%, *w*/*v*), 3 mL crude enzyme filtrate (corresponding to an enzyme loading of approximately 2.8 mg protein/g biomass), 0.3 mL 2% Prolin300 (as antimicrobial agent), and 26.7 mL citrate buffer (0.05 M, pH 4.8). The exact total protein concentration of each crude enzyme was determined by Bradford assay, and loadings were equalized by diluting the more concentrated sample with sterile buffer when necessary. In cases where loadings could not be matched (as originally designed), data were normalized to protein content post hoc. Incubation was performed at 50 °C, at 150 rpm. Samples (1 mL) were taken every 12 h, immediately boiled for 5 min to inactivate enzymes, centrifuged, and reducing sugars were measured by DNS [36].

### 2.8. Glucose Content Measurement

Glucose was quantified by modified glucose oxidase method [37].

### 2.9. RNA Extraction and Real-Time Quantitative Reverse Transcription-PCR (RT-qPCR) Analysis

Total RNA was extracted from mycelia (48 h) using TRIzol (Ambion™, Thermo Fisher Scientific, Waltham, MA, USA) as described [38,39,40]. One-step RT-qPCR was performed with SuperMix (TransGen Biotech Co., Ltd., Beijing, China). Relative expression was calculated by 2^−ΔΔCt^, with actin as internal control (primers used are listed in Table S5; RT-qPCR cycling conditions, such as: 50 °C 30 min; 94 °C 2 min; 40 cycles of 94 °C 15 s, 60 °C 30 s).

### 2.10. Statistical Significance Tests

Three biological and three technical replicates were used. Significance was determined by *t* test with FDR correction in Prism GraphPad 9.5.0.

### 2.11. Plate Assay of T. reesei in Medium with Oxidative Stress

Conidia were inoculated into liquid MM + 2% glucose in Petri dishes (static, 25 °C, constant light) until the exponential phase. Mycelial disks were transferred to PDA plates with 0, 5, or 10 mM H_2_O_2_. Growth diameter was measured after 72 h. Relative growth rate was calculated as described [41]. Plates were photographed when WT reached the plate edge.

### 2.12. Determination of Hydrogen Peroxide Concentration

Extracellular H_2_O_2_ was measured by titanium sulfate colorimetric method using a commercial kit. Briefly, culture supernatant (filtered through 0.22 μm) was mixed with acetone and titanium reagent, incubated for 5 min at RT. Absorbance at 415 nm was measured. Standard curve (0–3 μmol/mL H_2_O_2_) was used.

### 2.13. LC-MS/MS Protein Identification

Sample preparation: The distinctive ~80 kDa protein band was excised from Coomassie-stained SDS-PAGE gels. Gel slices were destained with 50% acetonitrile (ACN)/25 mM ammonium bicarbonate, reduced with 10 mM DTT at 56 °C for 45 min, alkylated with 55 mM iodoacetamide in the dark for 30 min, and digested overnight with trypsin (Promega Corporation, Madison, WI, USA, sequencing grade) at 37 °C. Peptides were extracted with 50% ACN/5% formic acid, dried by vacuum centrifugation, and resuspended in 0.1% formic acid.

LC-MS/MS analysis: Peptide mixtures were separated on an EASY-nLC 1200 system (Thermo Fisher Scientific, Waltham, MA, USA) coupled to a Q Exactive Plus hybrid quadrupole-Orbitrap mass spectrometer (Thermo Fisher Scientific, Waltham, MA, USA) equipped with a nano-electrospray ionization source. Samples were loaded onto a C18 trap column and separated on a C18 analytical column (15 cm × 75 µm i.d., 3 µm particles) with a linear gradient of 5–35% solvent B (0.1% formic acid in 80% ACN) over 60 min at a flow rate of 300 nL/min. The mass spectrometer was operated in a data-dependent acquisition mode, automatically switching between full MS (resolution [60,000 at 200 m/z], scan range 300–1500 m/z) and MS/MS fragmentation of the top 20 most abundant ions. HCD fragmentation was performed with a normalized collision energy of 28%.

Data analysis: Raw files were processed using Proteome Discoverer [PEAKS Studio] or MaxQuant against a combined database including *T. reesei* proteins (UniProt, taxonomy ID: 51453) and the *N. crassa cat-3* sequence, supplemented with common contaminants. Precursor mass tolerance was set to 10 ppm and fragment mass tolerance to 0.02 Da. Peptide identifications were filtered at a 1% FDR using the Percolator algorithm. Protein identification required at least two unique peptides. The top-ranked protein candidate was determined by four criteria: highest peptide count, highest average confidence score (−10lgP), largest total spectral area, and best comprehensive identification score.

## 3. Results

### 3.1. The Accumulation of Extracellular Hydrogen Peroxide Was Positively Correlated with the Production of T. reesei Cellulase During the Culture of Sugarcane Bagasse as Carbon Source

**Figure 1:**
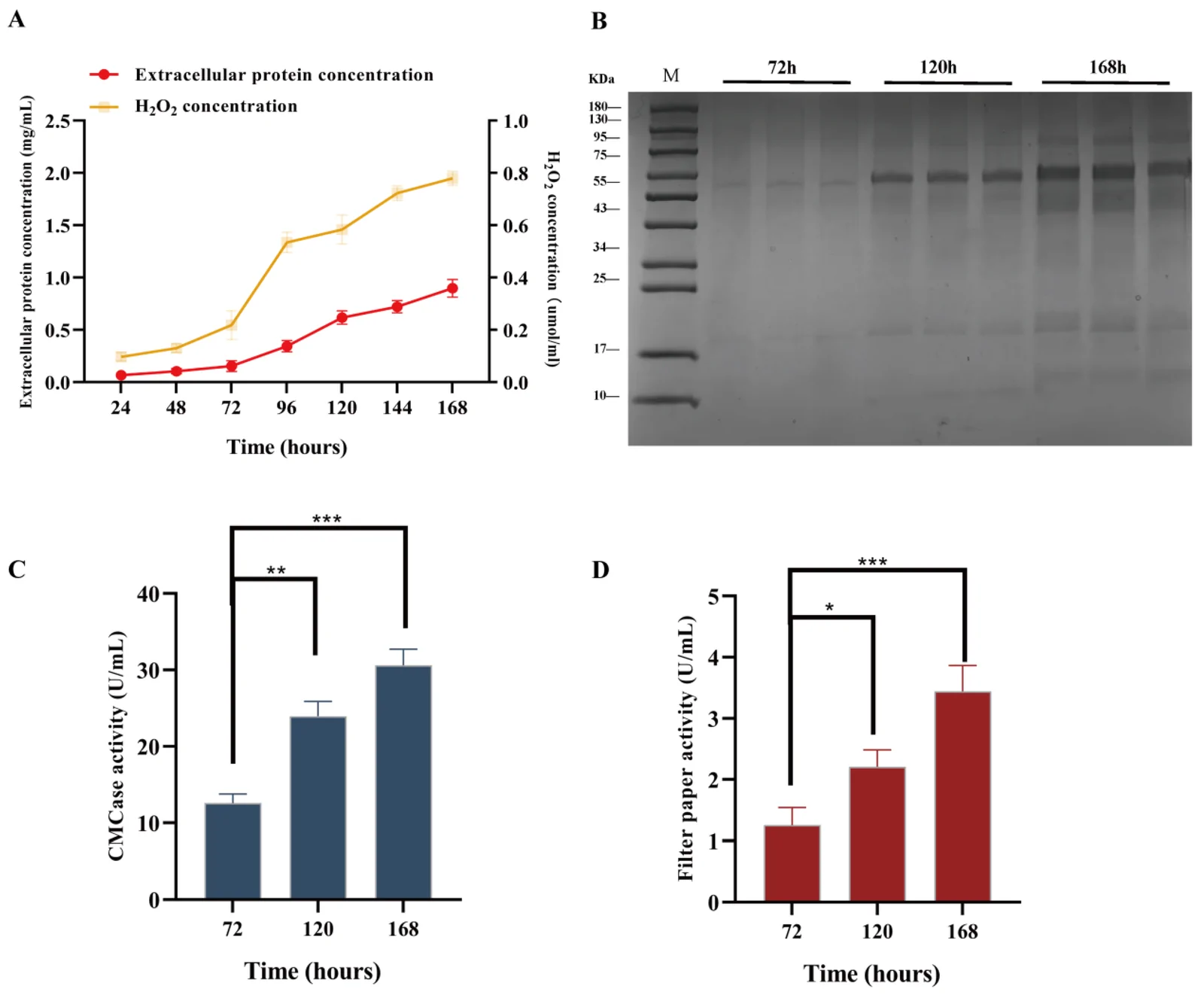
Expression of extracellular proteins, cellulase activity and hydrogen peroxide content in the supernatant of culture medium with bagasse as carbon source. (**A**) Line graph showing the 168 h extracellular protein expression levels of the *QM6a* strain versus hydrogen peroxide concentration in the culture supernatant. (**B**) SDS-PAGE analysis of the supernatant from *QM6a* cultured in a medium using sugarcane residue as carbon source. (**C**) The activity of CMCase in the supernatant of *QM6a* culture medium was analyzed by using bagasse as carbon source. (**D**) The activity of filter paper was analyzed in the supernatant of *QM6a* medium with bagasse as carbon source. The culture supernatant from the (**B**–**D**) was sampled at 168 h. The significance was based on *t* test analysis by the FDR approach. Asterisks indicate significant differences (*, *p* < 0.05; **, *p* < 0.01; ***, *p* < 0.001). ns, not significant.

To investigate the dynamic changes in H_2_O_2_ content during cellulase expression by *T. reesei*, the wild-type *QM6a* strain of *T. reesei* was used as the control in this experiment [42]. We cultured *QM6a* in MM medium using sugarcane bagasse as the carbon source for 168 h, measuring extracellular protein concentration and H_2_O_2_ levels in the supernatant every 24 h. Figure 1A shows that hydrogen peroxide levels remained stable from 0 to 24 h. From 48 to 96 h, H_2_O_2_ levels increased significantly with the rise in extracellular protein expression. Hydrogen peroxide levels continued to increase throughout the 96–168 h period, albeit at a diminishing rate. As shown in Figure 1A,B, extracellular protein concentration exhibited a sustained increase from 0 to 168 h, with the most pronounced accumulation occurring between 96 and 120 h, followed by continued progression until the endpoint. Consistent with this trend, the SDS-PAGE profile in Figure 1B reveals analogous extracellular protein expression patterns and distinct cellulase bands at ~75 kDa. CMCase and filter paper activity (FPase) were quantified in Figure 1C,D, respectively. When grown on sugarcane bagasse as the carbon source, *QM6a* demonstrated substantial CMCase and FPase activities at 168 h, confirming that its secreted cellulolytic enzymes effectively degraded both carboxymethyl cellulose and filter paper substrates. The synchronous elevation in these enzymatic activities correlated with the extracellular protein expression dynamics (Figure 1B–D). These data indicate that during the initial cultivation phase (0–24 h), even in the presence of cellulose, the microorganisms prioritize utilizing readily available carbon sources for rapid growth. At this stage, cellulose-degrading enzyme systems remain under-induced, with microbial metabolism focused on growth. H_2_O_2_, as a routine metabolic byproduct, is produced in low quantities and maintains dynamic equilibrium with cellular clearance mechanisms like catalase (Figure 1A). The rapid H_2_O_2_ accumulation phase (72–96 h) coincided with escalating extracellular protein levels. Subsequent moderate increases in both parameters persisted through 168 h, with protein showing marked elevation during 72–120 h (Figure 1A,B).

To quantitatively evaluate the relationship between H_2_O_2_ accumulation and cellulase production, linear regression analysis was performed on the data from 24 to 168 h (the period of active cellulase synthesis). A scatter plot of mean extracellular H_2_O_2_ concentration versus mean extracellular protein concentration showed a strong linear relationship (Table S1). The regression yielded a coefficient of determination R^2^ = [0.9892], confirming a significant positive linear correlation between these two variables.

The data demonstrates a linear positive correlation between H_2_O_2_ concentration and cellulase expression during *T. reesei* cultivation. Reducing H_2_O_2_ in the culture medium may improve the oxidative environment, thereby enhancing cellulase production.

### 3.2. Heterologous Expression of N. crassa Cat-3 in T. reesei Confers Enhanced Resistance to Oxidative Stress

**Figure 2:**
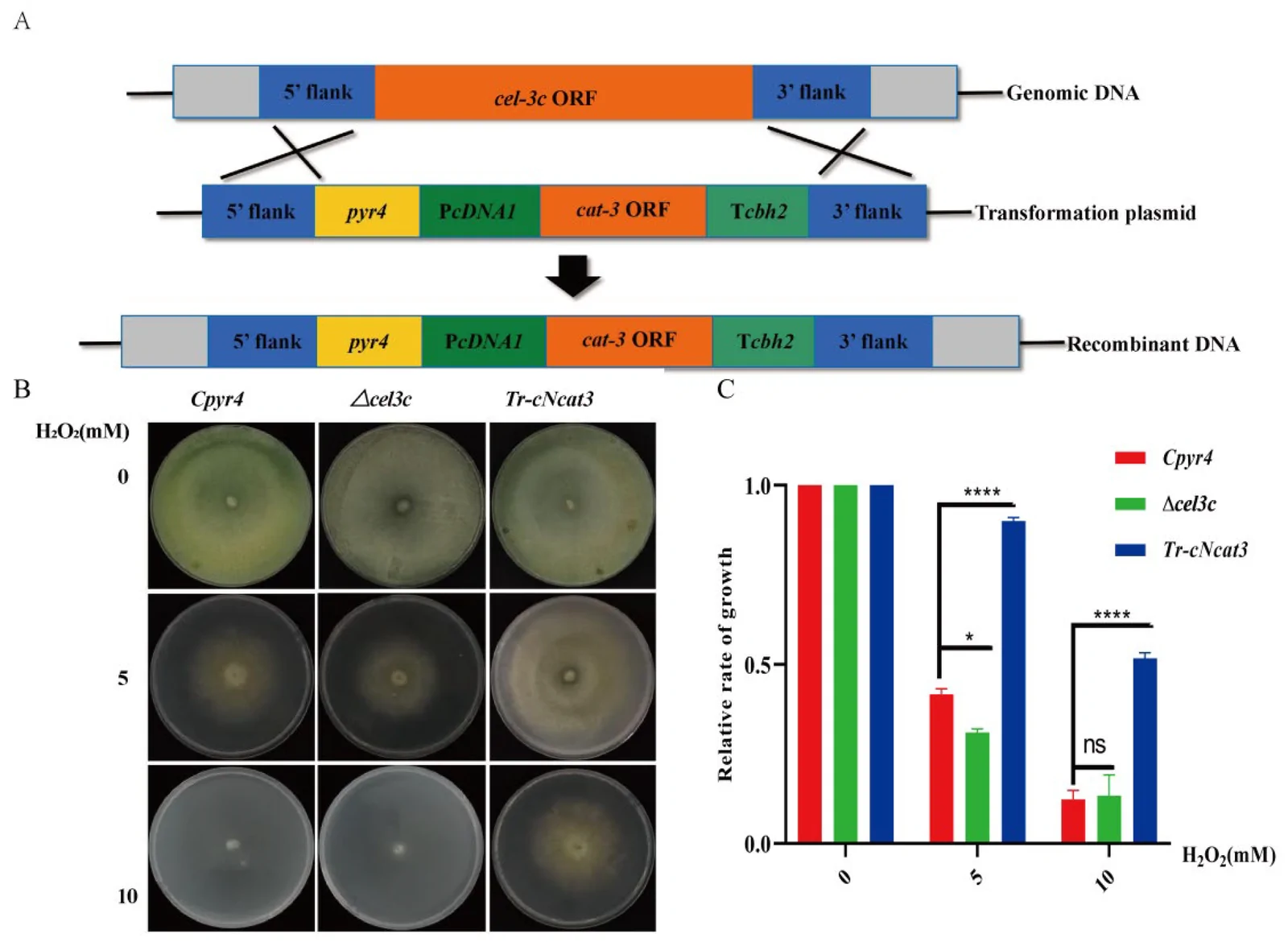
Construction of recombinant *Tr-cNcat3* and H_2_O_2_ resistance testing. (**A**) Schematic representation of the recombinant strain construction process. (**B**) Mycelial growth of *Cpyr4*, *Δcel3c*, and *Tr-cNcat3* under 0, 5, 10 mM H_2_O_2_ conditions. A suspension containing 1 × 10^5^ conidia of *T. reesei* was inoculated at the center of a plate and cultured for 72 h under constant conditions of 25 °C. (**C**) The relative growth rates of *Cpyr4*, *Δcel3c*, and *Tr-cNcat3* under different concentrations of H_2_O_2_. The growth diameter of each strain in 0 mM H_2_O_2_ medium was defined as the reference unit “1”. Relative growth rates were then calculated for strains in 5 mM and 10 mM H_2_O_2_ media. Data represent mean ± SD. Statistical significance was determined by two-way ANOVA followed by Tukey’s multiple comparison test. ns, not significant; * *p* < 0.05; **** *p* < 0.0001.

The gene *cat-3* is a catalase encoding gene that fundamentally breaks down the toxic byproduct H_2_O_2,_ generated by cellular metabolism, into harmless water and oxygen, serving as the core defense mechanism against oxidative stress [43]. To improve the oxygen supply and transport in the cultivation process of *T. reesei*, we engineered a recombinant strain through targeted gene replacement. The gene of *cat-3* ORF from *N. crassa* was homologously recombined into the *cel3c* locus of *T. reesei Tu6Δku70* (Figure 2A), generating the strain designated *Tr-cNcat3*. Notably, this genetic manipulation simultaneously addressed the uridine auxotrophy of the parental strain through *pyr4*-based complementation. For rigorous experimental controls, we constructed a parallel uridine complementary strain (C*pyr4*) by transforming *Tu6*Δ*ku70* with the pSK-*pyr4* vector [44]. Scrape the appropriate amount of conidia from transformed colonies of Trichoderma reesei after electroporation, extract their genomic DNA as a PCR verification template, and amplify using primer pairs P1 (Vcat3F/Vcel3cR) and P2 (V3cF/V3cR) (Table S5).

Positive strains showed a specific band at 1.6 kb (Figure 2B). As shown in the figure, the external primer pair P1 (Vcat3F/Vcel3cR) was used to verify whether the *cat-3* expression cassette was integrated into the cel3c gene locus of Trichoderma reesei, while the internal primer pair P2 (V3cF/V3cR) was used to verify whether the *cel3c* gene of Trichoderma reesei still existed. By combining the strategy of uracil auxotrophy and molecular verification, we successfully screened out positive recombinant strains of *T. reesei* transformed with the heterologous catalase gene, T1, T2, T4-T8 (Figure S1).

To verify the normal functioning of catalase activity, mycelial growth of *Cpyr4*, Tr-cNcat3, and *Δcel3c* was monitored in PDA plates supplemented with 0, 5, and 10 mM H_2_O_2_. As shown in Figure 2B,C, two-way ANOVA revealed significant main effects of strain (*p* < 0.0001) and H_2_O_2_ concentration (*p* < 0.0001), along with a highly significant interaction effect between the two factors (*p* < 0.0001). This indicates that the growth inhibitory response to H_2_O_2_ was strain-specific.

Post hoc analysis further clarified the specific differences: At 5 mM H_2_O_2_, *Tr-cNcat3* exhibited significantly higher relative growth rates compared to both *Cpyr4* and *cel3c* (*p* < 0.05). However, at 10 mM H_2_O_2_, while *Tr-cNcat3* maintained a significantly higher growth rate than Cpyr4 (*p* < 0.0001), there was no significant difference in growth between *Cpyr4* and *cel3c* (*p* > 0.05). All strains showed comparable growth in the absence of H_2_O_2_ (0 mM). To confirm that the enhanced hydrogen peroxide resistance conferred by *cat-3* expression is stable across independent transformation events, we tested three randomly selected transformants (*Tr-cNcat3-1*, *Tr-cNcat3-2*, *and Tr-cNcat3-3*) for hydrogen peroxide sensitivity. All three transformants exhibited the same antioxidant phenotype (Figure S2), ruling out clonal variation.

This indicates that *cat-3* is normally expressed and functional in the *Tr-cNcat3* strain, and suggests no significant correlation between *cel3c* gene deletion and hydrogen peroxide resistance. Taken together, this study successfully constructed a *Tr-cNcat3* strain with higher CAT-3 activity, offering a novel approach to mitigate peroxide accumulation during *T. reesei* growth.

### 3.3. Cat-3 Expression Boosts Total Cellulase Production and Specifically Enhances the Expression of Endogenous β-Glucosidase

**Figure 3:**
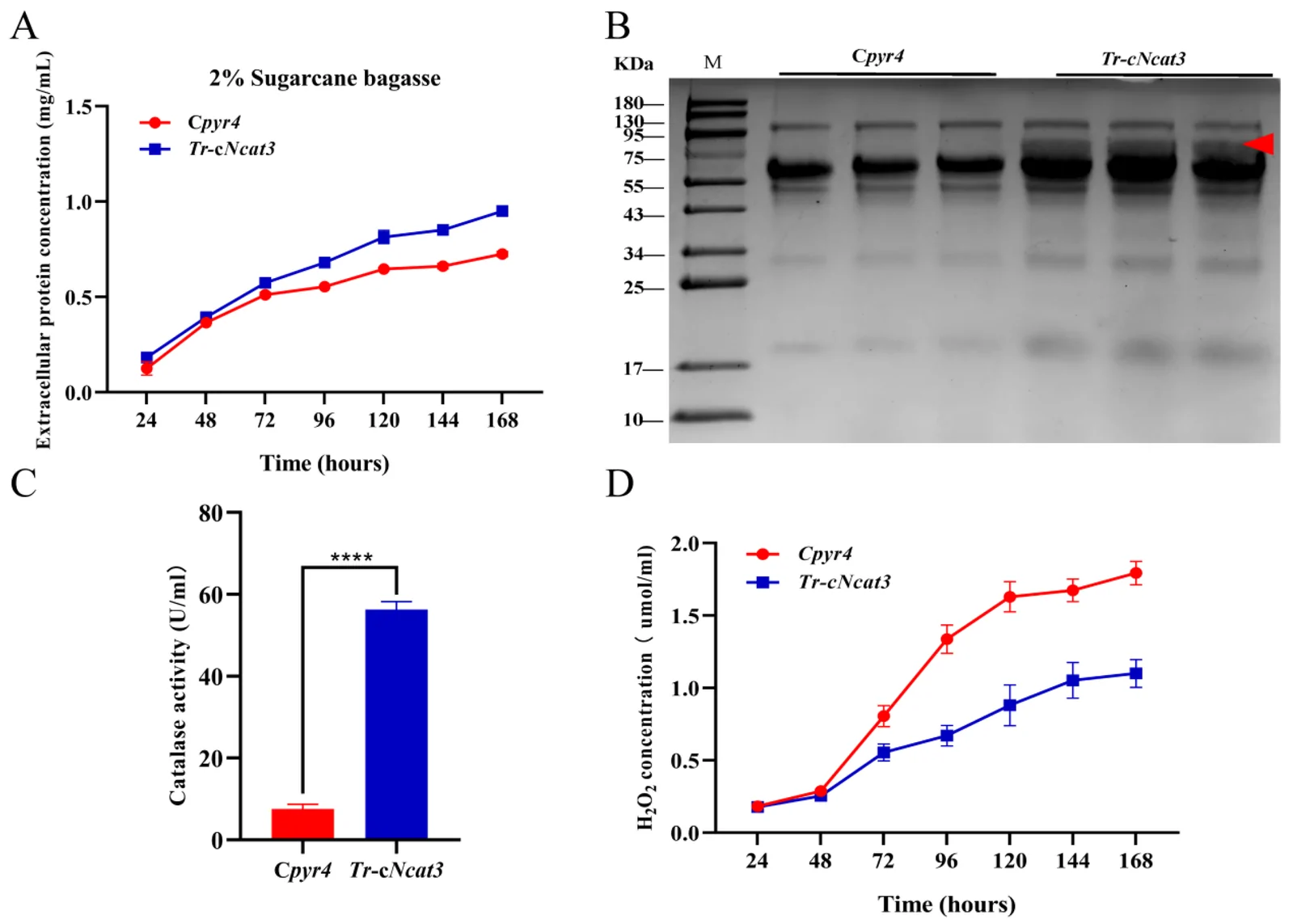
Comparison of *Tr-cNcat3* and *Cpyr4* cellulase expression levels with hydrogen peroxide concentration in the culture medium. (**A**) The total protein expression levels of *Cpyr4* and *Tr-cNcat3* when grown on sugarcane bagasse. (**B**) SDS-PAGE analysis of *Cpyr4* and *Tr-cNcat3* in the supernatant of culture medium. The red arrow indicates the position of the β-glucosidase protein band. (**C**) Comparative catalase activity of *Cpyr4* and *Tr-cNcat3* strains, measured in culture supernatants in sugarcane bagasse carbon source. (**D**) Determination of hydrogen peroxide content in supernatant of *Cpyr4* and *Tr-cNcat3* strains. The culture supernatant from the (**B**,**C**) was sampled at 168 h. The significance was based on *t* test analysis by the FDR approach. Asterisks indicate significant differences (****, *p* < 0.0001). ns, not significant.

To evaluate the potential enhancement of cellulase expression levels in *T. reesei* through *cat-3* expression, the *Cpyr4* and *Tr-cNcat3* strains were cultivated in MM using sugarcane bagasse as a carbon source. After 168 h, the supernatants were subjected to SDS-PAGE analysis and protein concentration assays. The data showed extracellular protein expression by *Tr-cNcat3* was slightly higher than *Cpyr4* in sugarcane bagasse (Figure 3A,B). To investigate whether the overall increase in *Tr-cNcat3* cellulase expression under bagasse culture conditions resulted from improved medium oxidation, we measured H_2_O_2_ content and catalase activity in the supernatants of *Cpyr4* and *Tr-cNcat3* cultures. As shown in Figure 3C,D, cellulase was significantly induced during the *Tr-cNcat3* cultivation using bagasse as the carbon source. The hydrogen peroxide levels in the medium supernatant also began to rise with the expression of cellulase, but remained significantly lower than those of the *Cpyr4* strain. *Tr-cNcat3* exhibited higher catalase activity in supernatants after 168 h compared to *Cpyr4*. This demonstrates that heterologous expression of *cat-3* in *Cpyr4* improved oxidative conditions in its growth environment, thereby enhancing cellulase production under bagasse culture conditions.

SDS-PAGE electrophoresis revealed an additional band at approximately 80 kDa in the *Tr-cNcat3* strain cultured in sucrose or rice straw-based media, as indicated by the red arrow in Figure 3B. To verify that the protein band at 80 kDa was not a random artifact, three independent transformants (*Tr-cNcat3-1*, *Tr-cNcat3-2*, *and Tr-cNcat3-3*) were cultured under identical fermentation conditions. As shown in Figure S3, all three transformants exhibited the same specific band at approximately 80 kDa.

This indicates that the heterologous expression the gene of *cat-3* not only increases the overall expression level of cellulase under the culture conditions using bagasse as the carbon source, but also alters the expression profile of its extracellular proteins.

### 3.4. Identification of β-Glucosidase as a Protein Upregulated by Cat-3 Overexpression via LC-MS/MS

**Figure 4:**
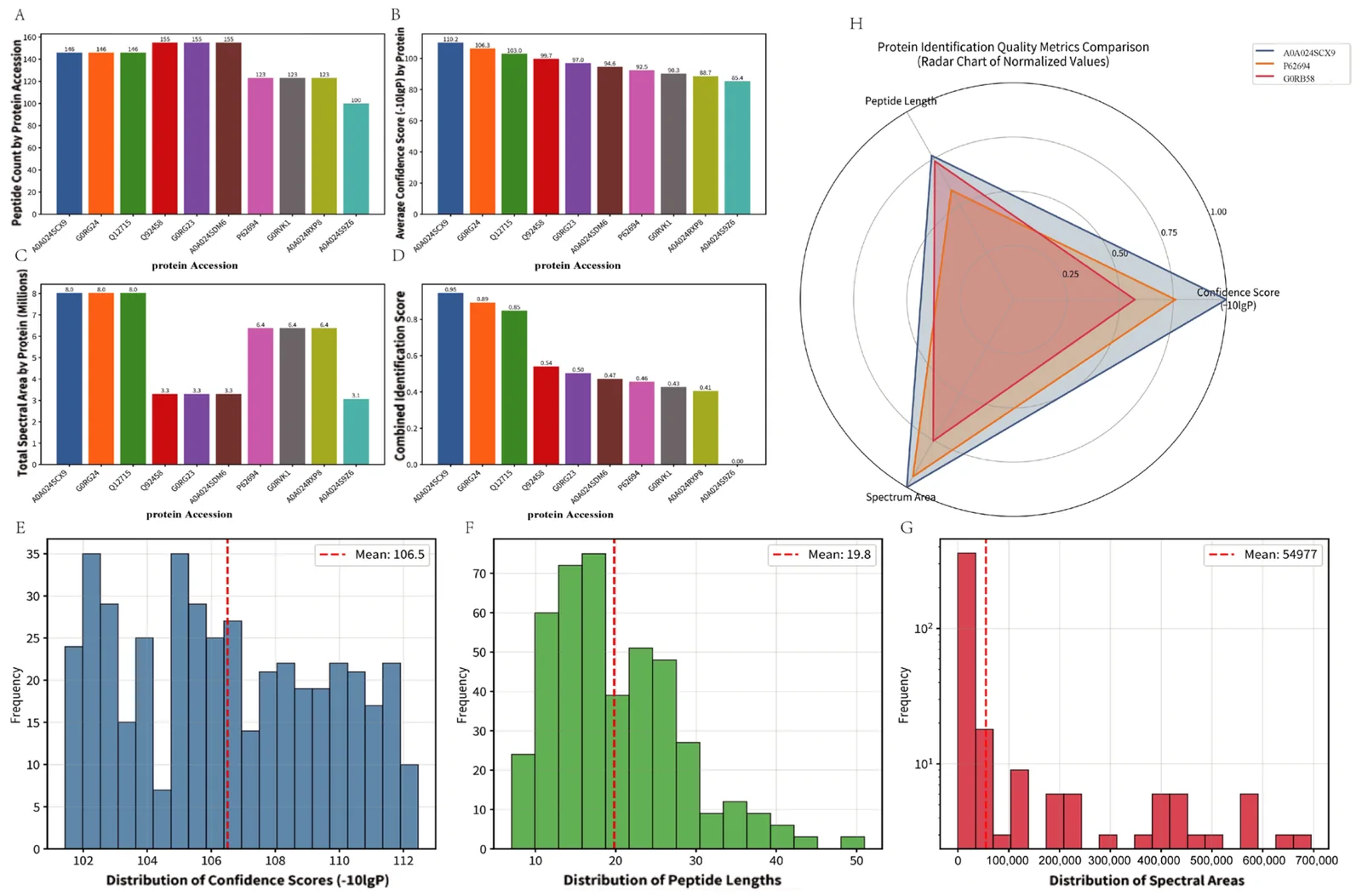
Mass spectrometry data analysis. (**A**–**D**) Comprehensive comparison of the top 10 protein candidates across four key metrics: (**A**) Peptide count (higher indicates more confident identification); (**B**) Average confidence score (−10lgP, higher indicates better quality matches); (**C**) Total spectral area (higher indicates greater abundance); (**D**) Combined identification score (normalized composite metric). (**E**,**F**) Detailed analysis of the top protein candidate (A0A024SCX9) showing distributions of: (**E**) Confidence scores (−10lgP); (**F**) Peptide lengths; (**G**) Spectral areas. (**H**) Radar chart comparing the top 3 protein candidates across three normalized metrics, providing an intuitive visualization of relative performance.

To identify the specific protein that was added as a band, the band was subjected to LC-MS/MS analysis. Conventional screening based on multiple criteria identified the protein with accession number A0A024SCX9 as the top-ranked candidate. As shown in Figure 4A–D, the ten highest-ranking proteins for each of four key identification metrics were selected: the highest number of peptides, the highest average confidence score, the largest total spectral area, and the best comprehensive identification score. The protein A0A024SCX9 ranked first across all four criteria. To rigorously validate this candidate as the correctly identified protein, a detailed analysis of its constituent peptides was performed, including their confidence scores, lengths, and spectral areas (Figure 4E–G). A radar chart comparing the normalized performance of the top three candidates for these three metrics (confidence score, peptide length, and spectral area) further confirmed the strong performance of A0A024SCX9, which occupied the largest area on the chart, indicating the highest likelihood of being the correct identification (Figure 4H). Table S2 summarizes the candidate proteins ranked by their comprehensive identification score. Table S2 clearly shows that candidate A0A024SCX9 held the top position for all four metrics (highest peptide count, highest average confidence score, largest total spectral area, and best comprehensive score). Subsequently, a search against the UniProt database using this accession number confirmed the protein’s identity as β-glucosidase (CEL3A). The raw data of the mass spectrometry are available in Supplementary Materials Tables S3 and S4. To determine whether the β-glucosidase band formation correlates with *cel3c* gene deletion, SDS-PAGE analysis of *Δcel3c* strains cultured with bagasse revealed the absence of the red arrow-marked β-glucosidase protein band. This suggests that β-glucosidase upregulation is linked to *cat-3* overexpression rather than *cel3c* deletion, although contributions from other genetic background effects (e.g., locusspecific effects, secondary mutations) cannot be completely excluded without an empty vector control (Figure S4).

### 3.5. The Impaction of β-Glucosidase by Cat-3 Is Dependent on Lignocellulosic Carbon Sources

**Figure 5:**
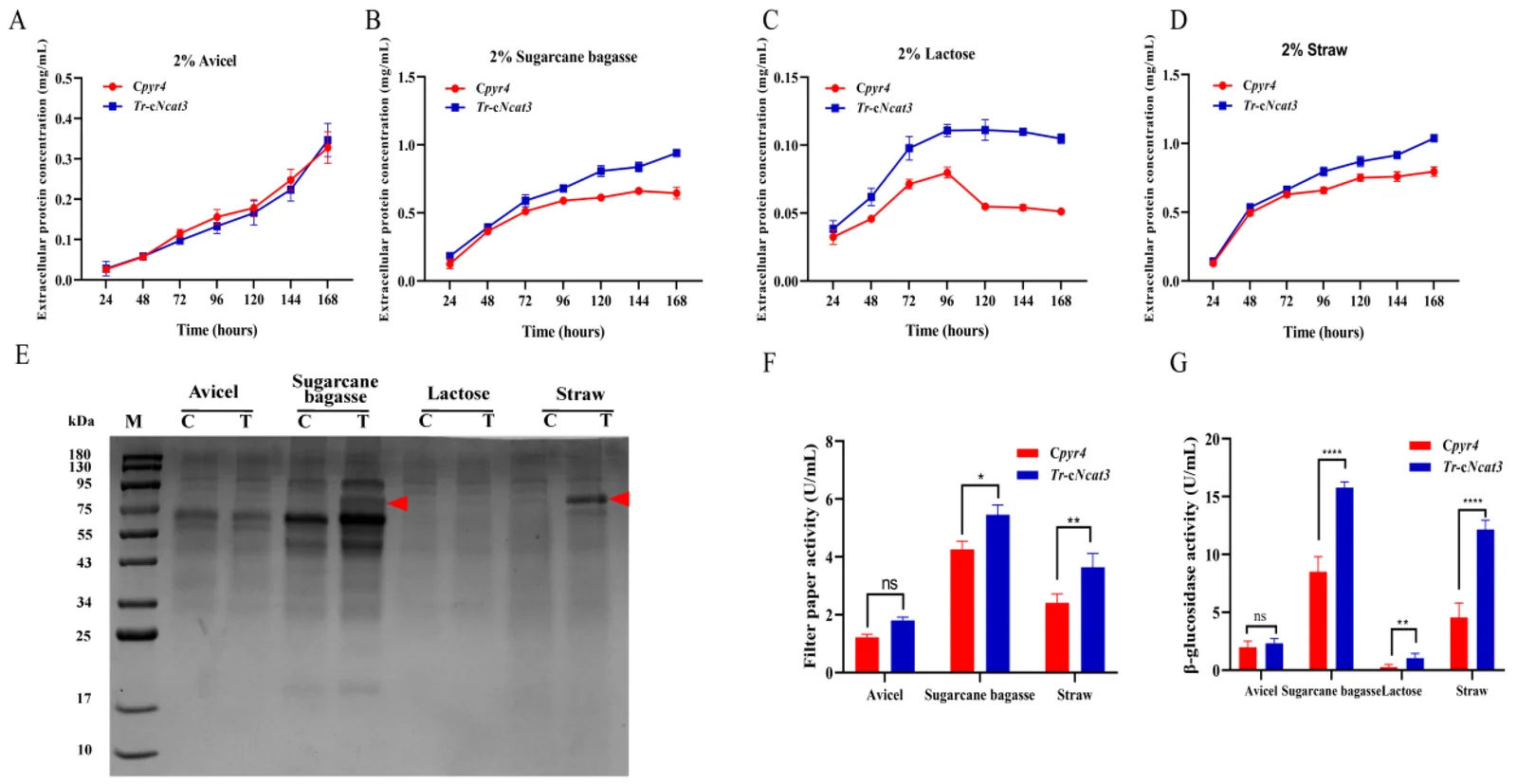
Impact of *cat-3* expression on *T. reesei* cellulase expression among different carbon sources. (**A**–**D**) The total protein expression levels of *Cpyr4* and *Tr-cNcat3* when grown on Avicel (**A**), sugarcane bagasse (**B**), lactose (**C**), and straw (**D**), as carbon sources. (**E**) SDS-PAGE analysis of the (**A**–**D**) supernatant from the culture medium at 168 h. The red arrow indicates the position of the β-glucosidase protein band. (**F**,**G**) The filter paper activities (**F**) and β-glucosidase activities (**G**) in the same supernatants as in (**A**–**D**) at 168 h. Data represent mean ± SD. Statistical significance was determined by two-way ANOVA followed by Tukey’s multiple comparison test. ns, not significant; (*, *p* < 0.05; **, *p* < 0.01; ****, *p* < 0.0001).

Carbon sources are a critical factor for microbial growth. To investigate why heterologous expression of *cat-3* enhances the overall level of cellulase expression and specifically boosts the expression of β-glucosidase, this study cultured *Tr-cNcat3* and *Cpry4* in MM medium and employed four carbon sources: bagasse, straw, Avicel, and lactose. As shown in Figure 5A–E, *Tr-cNcat3* exhibited higher cellulase expression than *Cpry4* under sugarcane bagasse, straw, and lactose conditions. As illustrated in Figure 4A–D, the extracellular protein expression levels of *Cpyr4* and *Tr-cNcat3* were comparable when Avicel was used as the carbon source. When cultured on bagasse, straw, or lactose, *Tr-cNcat3* exhibited higher extracellular protein production than *Cpyr4*. As shown in Figure 5E, high β-glucosidase protein expression by *Tr-cNcat3* was observed only when straw or bagasse served as the carbon sources. Regarding filter paper activity (FPA) (Figure 5F), two-way ANOVA revealed significant main effects of strain and carbon source on both filter paper activity (FPA) and β-glucosidase activity (all *p* < 0.0001). For FPA, no significant strain–substrate interaction was detected (*p* = 0.0715), indicating a consistent strain improvement across carbon sources; post hoc analysis confirmed that *Tr-cNcat3* exhibited significantly higher FPA than Cpyr4 on bagasse and Avicel (*p* < 0.01 and *p* < 0.05, respectively), but not on straw. The determination of filter paper activity is based on the detection of reducing sugars. As lactose itself is a reducing sugar, conventional assays measuring reducing sugar concentration are rendered unsuitable for quantifying filter paper activity in the culture supernatant. In contrast, β-glucosidase activity showed a highly significant strain–substrate interaction (*p* < 0.0001), reflecting substrate-specific enhancement (Figure 5G); *Tr-cNcat3* was superior to *Cpyr4* on bagasse, straw, and lactose (*p* < 0.01 or *p* < 0.0001), whereas no significant difference was observed on Avicel.

The results demonstrated that, compared to *Cpyr4*, the *Tr-cNcat3* strain enhanced cellulase expression across three carbon sources (bagasse, straw, and lactose), with specific enhancement of β-glucosidase expression under bagasse and straw conditions. This suggests that *cat-3* does not directly activate β-glucosidase expression but rather exerts its regulatory function through specific physiological stress triggered by lignocellulosic substrates.

### 3.6. Organic Nitrogen Sources Synergize with Cat-3 to Further Enhance Cellulase Production and β-Glucosidase Expression

To investigate whether other major nutritional signals (particularly nitrogen sources) besides carbon source signals are associated with the increased expression of cellulase and the specific enhancing of β-glucosidase in the *Tr-cNcat3* strain, we systematically studied the effects of different nitrogen sources. Building on experimental results demonstrating bagasse as the optimal carbon source, we further evaluated the effects of various nitrogen sources on cellulase system expression in *Tr-cNcat3* strains.

**Figure 6:**
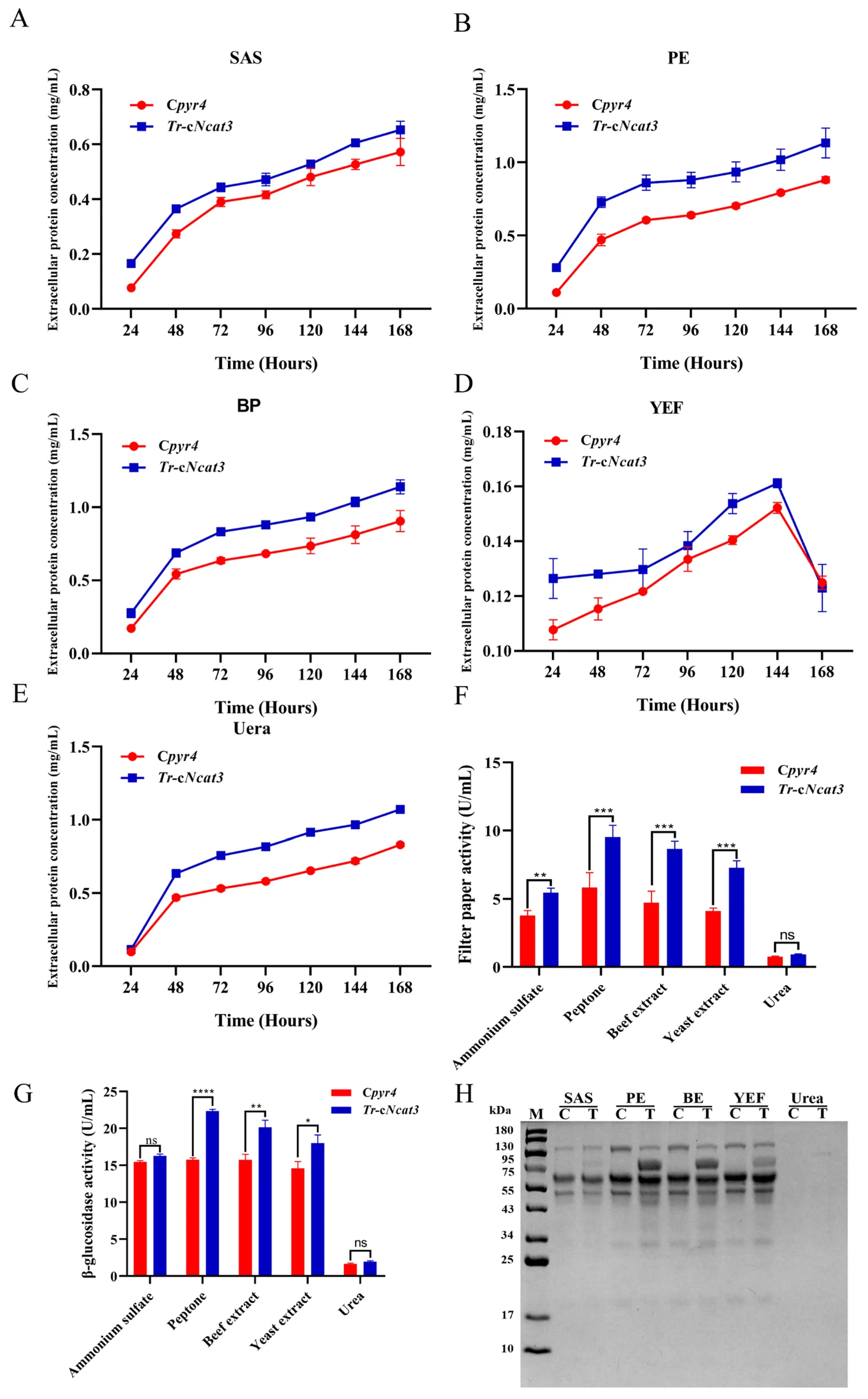
Impact of *cat-3* expression on *T. reesei* cellulase expression among different nitrogen sources. (**A**–**E**) The total protein expression levels of *Cpyr4* and *Tr-cNcat3* when grown on ammonium sulfate (**A**), peptone (**B**), beef extract (**C**), yeast extract (**D**) and urea (**E**) as nitrogen sources. (**F**,**G**) The filter paper activities (**F**) and β-glucosidase activities (**G**) in the same supernatants as in (**A**–**E**) at 168 h. H: SDS-PAGE analysis of the (**A**–**E**) supernatant from the culture medium at 168 h. (**H**): SDS-PAGE analysis the total protein expression levels of *Cpyr4* and *Tr-cNcat3* when grown on ammonium sulfate, peptone, beef extrac, yeast extract and ure as nitrogen sources The red arrow indicates the position of the β-glucosidase protein band. Data represent mean ± SD. Statistical significance was determined by two-way ANOVA followed by Tukey’s multiple comparison test. ns, not significant; (*, *p* < 0.05; **, *p* < 0.01; ***, *p* < 0.001; ****, *p* < 0.0001).

We investigated different nitrogen sources affect protein expression in the recombinant *Tr-cNcat3* strain. The strains *Cpyr4* and *Tr-cNcat3* were cultured in MM supplemented with sugarcane bagasse and 0.5% peptone, beef extract, yeast extract, or urea as nitrogen sources, with ammonium sulfate (inorganic nitrogen control) as a reference. Extracellular protein expression, FPase, and β-glucosidase activity were measured. Compared to *Cpyr4*, *Tr-cNcat3* showed significantly higher protein expression, FPase, and β-glucosidase activity when grown with peptone, beef extract, and yeast extract (but not urea) (Figure 6A–G). Notably, the 80 kDa band (corresponding to endogenous β-glucosidase) exhibited increased intensity in *Tr-cNcat3* (Figure 6H), indicating that the combination of nitrogen supplementation and catalase expression further promoted β-glucosidase expression levels. Peptone was the most effective nitrogen source for the expression of extracellular proteins, the enhancement of filter paper activity and the β-glucosidase activity of *Tr-cNcat3* (Figure 6A–H).

This recombinant strain achieved a extracellular protein concentration of 1.13 mg/mL representing a 21.5% increase over *Cpyr4* (0.93 mg/mL) (Figure 6B). The enzymatic activities were similarly elevated, with FPase activity reaching 9.52 U/mL (63.6% higher than *Cpyr4*’ s 5.82 U/mL) and β-glucosidase activity attaining 22.35 U/mL (41.8% greater than *Cpyr4*’ s corresponding 15.76 U/mL) (Figure 6F,G).

### 3.7. RT-qPCR Analysis Reveals Cat-3-Mediated Reprogramming of the Cellulase Gene Expression Profile

**Figure 7:**
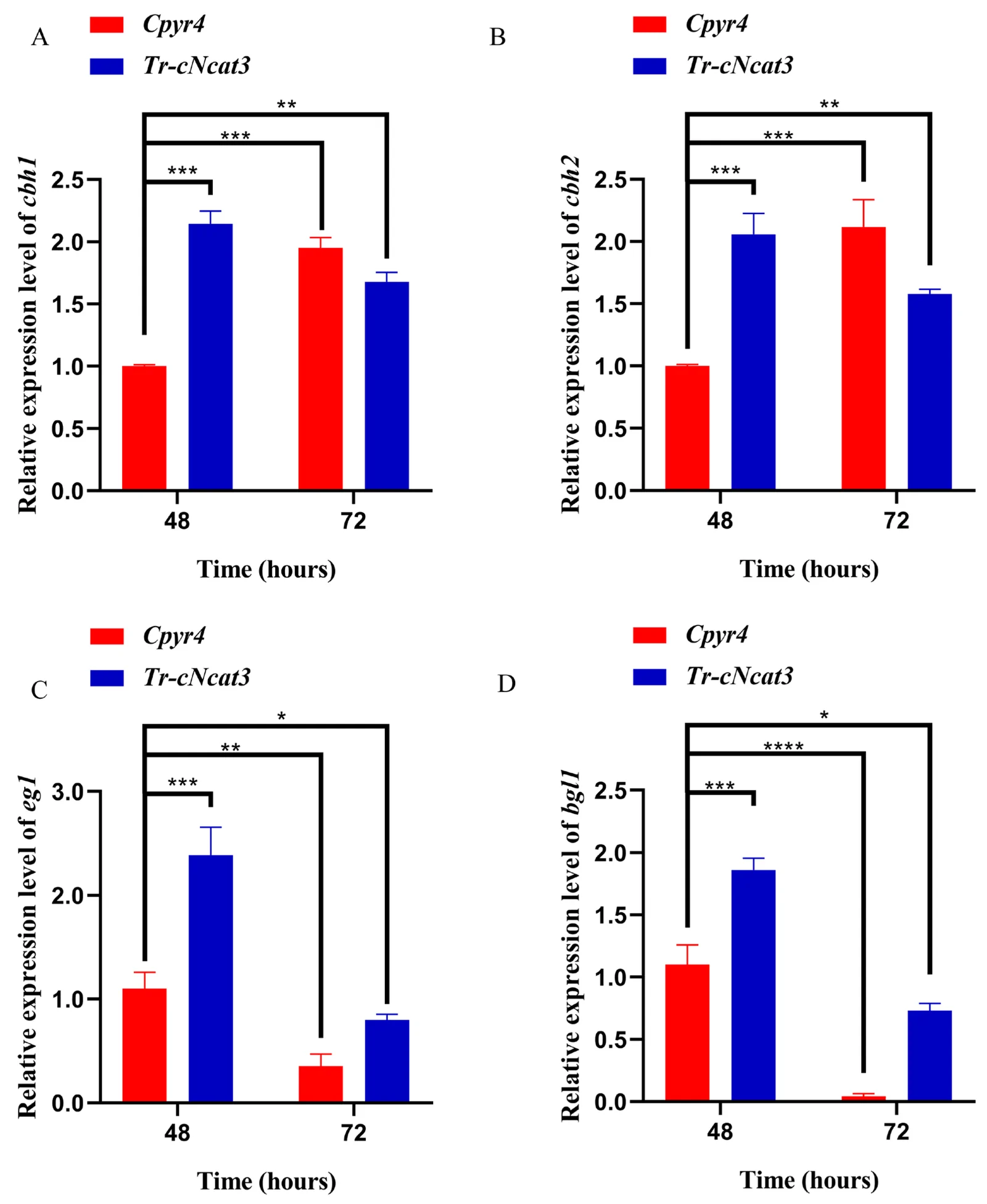
Transcriptional levels of key cellulase genes in *Cpyr4* and *Tr-cNcat3*. (**A**–**D**) The expression levels of *cbh1* (**A**), *cbh2* (**B**), *eg1* (**C**), and *bgl1* (**D**) were quantified by RT-qPCR in minimal medium containing sugarcane bagasse as carbon source and peptone as nitrogen source. The transcript levels of the *cbh1* (**A**), *cbh2* (**B**), *eg1* (**C**), and *bgl1* genes in the *Cpyr4* and strains were assessed at 48 and 72 h post-inoculation with an equal number of conidia. Transcription level of actin was used as the endogenous control. The values show the mean of three biological replicates, and the error bar indicates the standard deviation. Data represent mean ± SD. Statistical significance was determined by Two-way ANOVA followed by Tukey’s multiple comparison test (*, *p* < 0.05; **, *p* < 0.01; ***, *p* < 0.001; ****, *p* < 0.0001). ns, not significant.

To investigate whether the expression of *cat-3* affects the expression of the cellulase system at the transcriptional level in T. reesei, we analyzed the transcriptional dynamics of core cellulase genes (*cbh1*, *cbh2*, *eg1*, and *bgl1*) in the parental strain *Cpyr4* and the engineered strain *Tr-cNcat3* at two cultivation stages (48 h and 72 h) using RT-qPCR. Heterologous expression of *cat-3* significantly altered the cellulase gene expression profile in a temporally specific manner. (Two-way ANOVA revealed a significant strain × time interaction for *cbh1* (F(1,8) = 256.8, *p* < 0.0001), *cbh2* (F(1,8) = 97.14, *p* < 0.0001), and *eg1* (F(1,8) = 18.91, *p* = 0.0024), indicating that the transcriptional responses of these genes to the *cat-3* background differed across the two time points. In contrast, no interaction was observed for *bgl1* (*p* = 0.5261), while the main effects of strain (*p* < 0.0001) and time (*p* < 0.0001) were both significant.) While *Tr-cNcat3* exhibited early transcriptional upregulation of cbh1 and *cbh2* at 48 h compared to *Cpyr4*, this phenotype reversed at 72 h, when transcription levels declined in the *Tr-cNcat3* strain but increased in *Cpyr4* (Figure 7A,B). (This crossover pattern was statistically supported by the significant interactions, confirming that the temporal trajectories of *cbh1* and *cbh2* expression are fundamentally different between the two strains.)This biphasic response suggests that *cat-3* may boost early exoglucanase production. However, accelerated carbon metabolism in *Tr-cNcat3* could deplete sugar availability by 72 h, triggering carbon catabolite repression and dampening cellulase expression.

Unlike *cbh1* and *cbh2*, *eg1* and *bgl1* exhibited sustained transcriptional dominance in the *Tr-cNcat3* strain. The transcription levels of these genes in *Tr-cNcat3* surpassed those of *Cpyr4* at 48 h (Figure 7C,D). Both strains exhibited a decline in *bgl1* and *eg1* transcription levels at 72 h, (but the underlying dynamics differed. For *eg1*, the significant interaction (*p* = 0.0024) reflected a larger between-strain difference at 48 h (mean Δ ≈ 1.29) than at 72 h (mean Δ ≈ 0.44), indicating that the decline in *eg1* expression was more pronounced in Tr-cNcat3, even though its transcript levels remained higher than those in *Cpyr4* at both time points. For *bgl1*, however, the absence of a significant interaction corroborates that the superior expression in *Tr-cNcat3* was maintained consistently over time, with both strains showing a parallel decline from 48 to 72 h). Notably, *bgl1* expression in *Tr-cNcat3* remained significantly higher than in the *Cpyr4* strain, consistent with our previously observed *cat-3*-specific enhancement of β-glucosidase expression (Figure 3B).

The RT-qPCR data (together with the statistical results) revealed a sophisticated, biphasic response orchestrated by *cat-3* expression (Figure 7A–D). The early upregulation of exoglucanase genes (*cbh1*, *cbh2*) at 48 h in *Tr-cNcat3* suggests a strategy to rapidly initiate cellulose deconstruction, potentially fueled by an optimized redox state. The significant interactions for these genes confirm that this early advantage waned by 72 h, with a clear reversal in expression hierarchy. In stark contrast, the transcription levels of *eg1* and *bgl1* remained consistently high throughout the cultivation process, with *Tr-cNcat3* maintaining dominance over the *Cpyr4* strain. Although the transcription levels of eg1 and bgl1 in both strains declined after 72 h—a phenomenon consistent with the literature reports on the typical upregulation followed by down-regulation of *eg1* and *bgl1*—(the statistically unchanged advantage for *bgl1* across time points reinforces the specificity and robustness of *cat-3*-mediated β-glucosidase enhancement). Exogenous peptone addition further accelerated this process [45].

### 3.8. Crude Enzyme Broth from the Tr-cNcat3 Strain Show Enhanced Saccharification Efficiency on Lignocellulosic Biomasses

**Figure 8:**
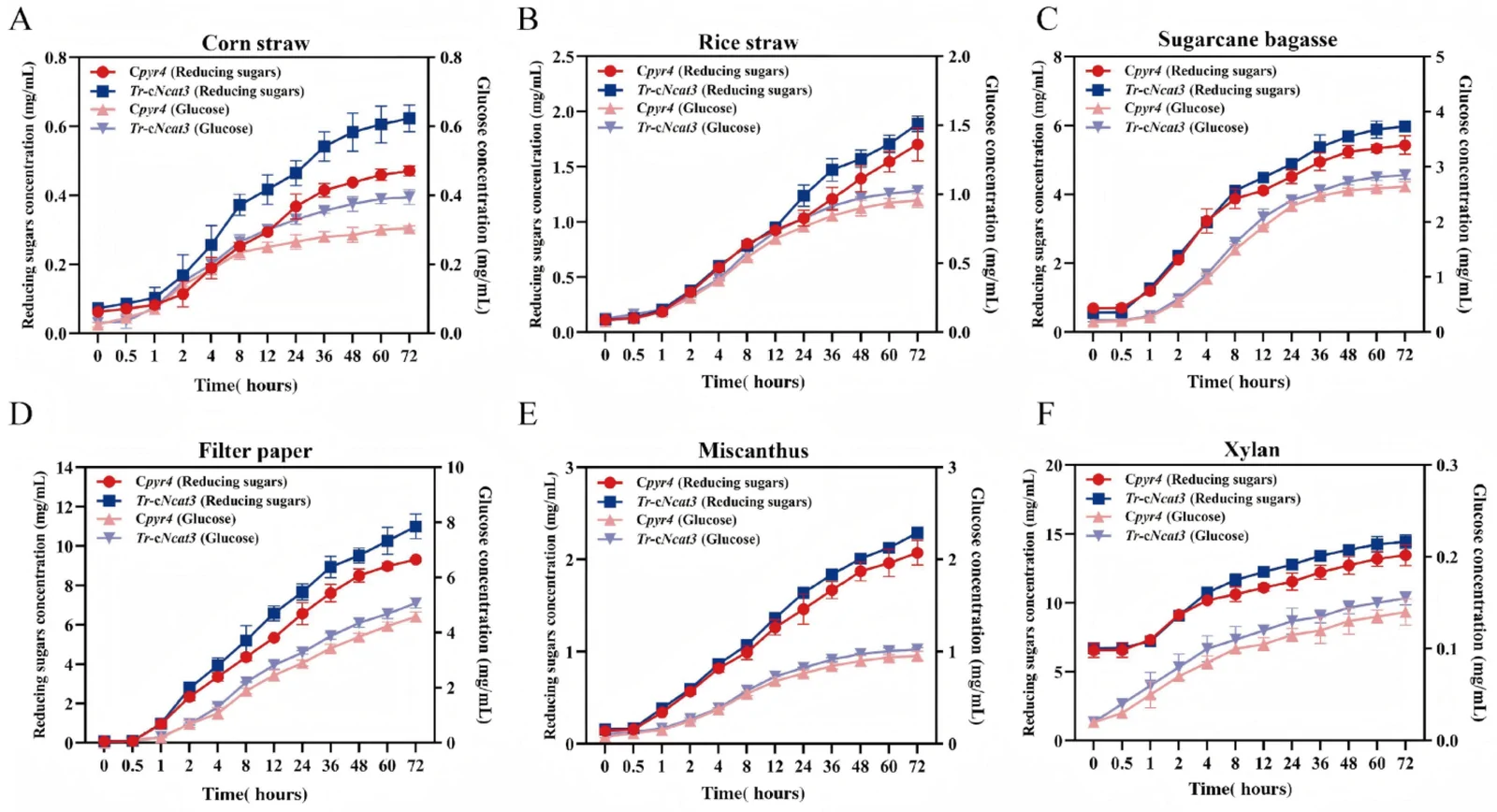
Lignocellulose degradation capabilities of *Cpyr4* and *Tr-cNcat3*. (**A**–**F**) The reducing sugar and glucose concentrations in the hydrolysates of corn straw (**A**), rice straw (**B**), sugarcane bagasse (**C**), filter paper (**D**), miscanthus (**E**), and xylan (**F**) by the supernatant from *Cpyr4* and *Tr-cNcat3* cultured in minimal medium (sugarcane bagasse as carbon source; peptone as nitrogen source).

To comprehensively assess the impact of *cat-3* expression on saccharification efficiency, building upon the optimized cultivation conditions (with sugar bagasse as carbon source and peptone as nitrogen source), we evaluated the lignocellulose degradation capacity of recombinant strain *Tr-cNcat3* against six representative substrates: corn straw, rice straw, sugarcane bagasse, filter paper, Miscanthus, and xylan. Using undiluted crude enzyme broth from both *Cpyr4* and *Tr-cNcat3*, we conducted comprehensive hydrolysis assays while monitoring reducing sugar and glucose release to elucidate the impact of *cat-3* expression on lignocellulose depolymerization kinetics. The recombinant strain *Tr-cNcat3* demonstrated markedly improved hydrolysis efficiency. Most notably, *Tr-cNcat3* crude enzyme yielded significantly higher reducing sugar and glucose from corn straw compared to *Cpyr4* (Figure 8A). Modest but consistent improvements were also observed for other substrates, with hydrolysis products from rice straw, sugarcane bagasse, filter paper, Miscanthus, and xylan all showing slight increases relative to *Cpyr4* (Figure 8B–F).

The industrial application of *T. reesei* has long been limited by insufficient β-glucosidase expression, which not only restricts cellulose degradation efficiency but also affects its economic feasibility [46]. This study demonstrates that overexpression of the *cat-3* gene can systematically optimize the cellulase system of this fungus: in the recombinant strain *Tr-cNcat3*, the expression of cellulases was enhanced through redox homeostasis regulation and secretion pathway optimization, particularly a significant increase in β-glucosidase expression, thereby improving lignocellulose degradation efficiency. These findings deepen our understanding of enzymatic regulation in filamentous fungi and significantly enhance the industrial potential of *T. reesei* for biomass conversion.

## 4. Conclusions

*T. reesei* is a premier industrial workhorse for cellulase production, yet its fermentation efficiency is often constrained by oxidative stress induced by oxygen limitation during large-scale cultivation. To mitigate this bottleneck, we engineered *T. reesei* to heterologously express the robust catalase gene *cat-3* from *N. crassa*. The engineered strain *Tr-cNcat3* exhibited enhanced antioxidant capacity, resulting in significantly reduced extracellular H_2_O_2_ accumulation. Concomitantly, extracellular protein secretion and total cellulase activities (including filter paper activity and β-glucosidase activity) were elevated. Notably, β-glucosidase—a rate-limiting component of the T. reesei cellulase system—showed dramatically enhanced production, particularly on lignocellulosic substrates such as bagasse and straw. This enzymatic enhancement was further potentiated by preferred organic nitrogen sources, ultimately translating to improved saccharification efficiency of agricultural residues using crude enzyme extracts. RT-qPCR analyses revealed that *cat-3* expression significantly altered the transcriptional profile of cellulase genes, suggesting that the observed changes are linked to redox homeostasis and nutrient signaling, rather than reflecting a simple global upregulation. These results demonstrate that heterologous catalase expression is an effective strategy for optimizing the *T. reesei* cellulase system, primarily through oxidative stress alleviation with concomitant β-glucosidase reinforcement. This approach not only enhances the industrial potential of *T. reesei* for cost-effective biorefining, but also sheds light on the interplay between redox balance and enzyme biosynthesis.

## Data Availability

The raw mass spectrometry data were generated at the Biomedical Instrumentation Sharing Platform, School of Life Sciences, Xiamen University, and are not publicly available due to the platform’s data management policy and service agreement restrictions. However, the source data supporting the findings of this study, including the search results used to generate Figure 4A–H, are provided in the Supplementary Materials (Supplementary File [Tables S1 and S2], Excel format).

## Supplementary Materials

The following supporting information can be downloaded at: https://www.mdpi.com/article/10.3390/jof12080554/s1, Figure S1: Validation results of T-CAT3 homokaryon; Figure S2:Growth rates of the strain on plates with different hydrogen peroxide concentrations; Figure S3: SDS-PAGE analysis of *Cpyr4* and *Tr-cNcat3-1*, *Tr-cNcat3-2*, *Tr-cNcat3-3* in the supernatant of culture medium. The red arrow indicates the position of the β-glucosidase protein band; Figure S4: SDS-PAGE analysis of Δcel3c and Tr-cNcat3 in the supernatant of culture medium. The red arrow indicates the position of the β-glucosidase protein band; Table S1: Linear regression table of protein concentration and hydrogen peroxide concentration; Table S2: Top protein candidates ranked by combined identification score; Table S3: Differential expression levels of cellulase-related genes under carbon sources; Table S4: Enzymatic activity of cellulases secreted by wild-type and mutant strains; Table S5: Primer sequences of major transcription factors related to cellulase production.
