# Supplementary material for: Heterologous Expression of a Neurospora crassa Catalase Reprograms the Cellulase System and Enhances β-Glucosidase Production in Trichoderma reesei
Source: J Fungi (Basel). 2026 Jul 25;12(8):554. doi: 10.3390/jof12080554 (PMC13514374; doi:10.3390/jof12080554)
Supplement: Supplementary file 1 [file jof-12-00554-s001.zip › Figsure S1.pdf]

Figsure. S1

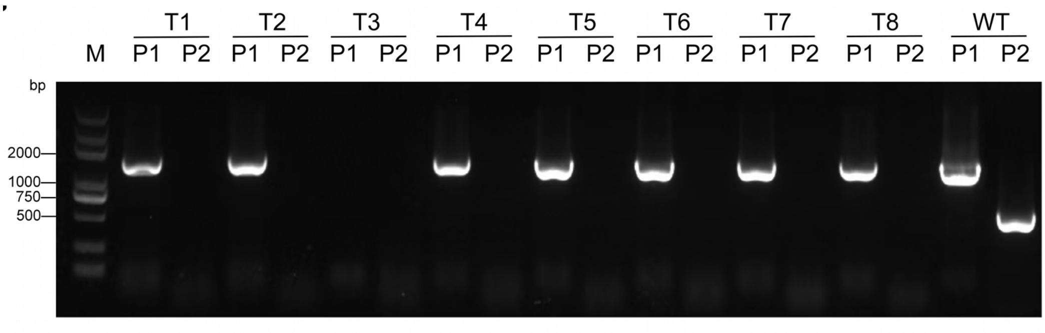

Validation results of T-CAT3 homokaryon.
