# Supplementary material for: Heterologous Expression of a Neurospora crassa Catalase Reprograms the Cellulase System and Enhances β-Glucosidase Production in Trichoderma reesei
Source: J Fungi (Basel). 2026 Jul 25;12(8):554. doi: 10.3390/jof12080554 (PMC13514374; doi:10.3390/jof12080554)
Supplement: Supplementary file 1 [file jof-12-00554-s001.zip › Figsure S2.pdf]

Figsure. S2

A

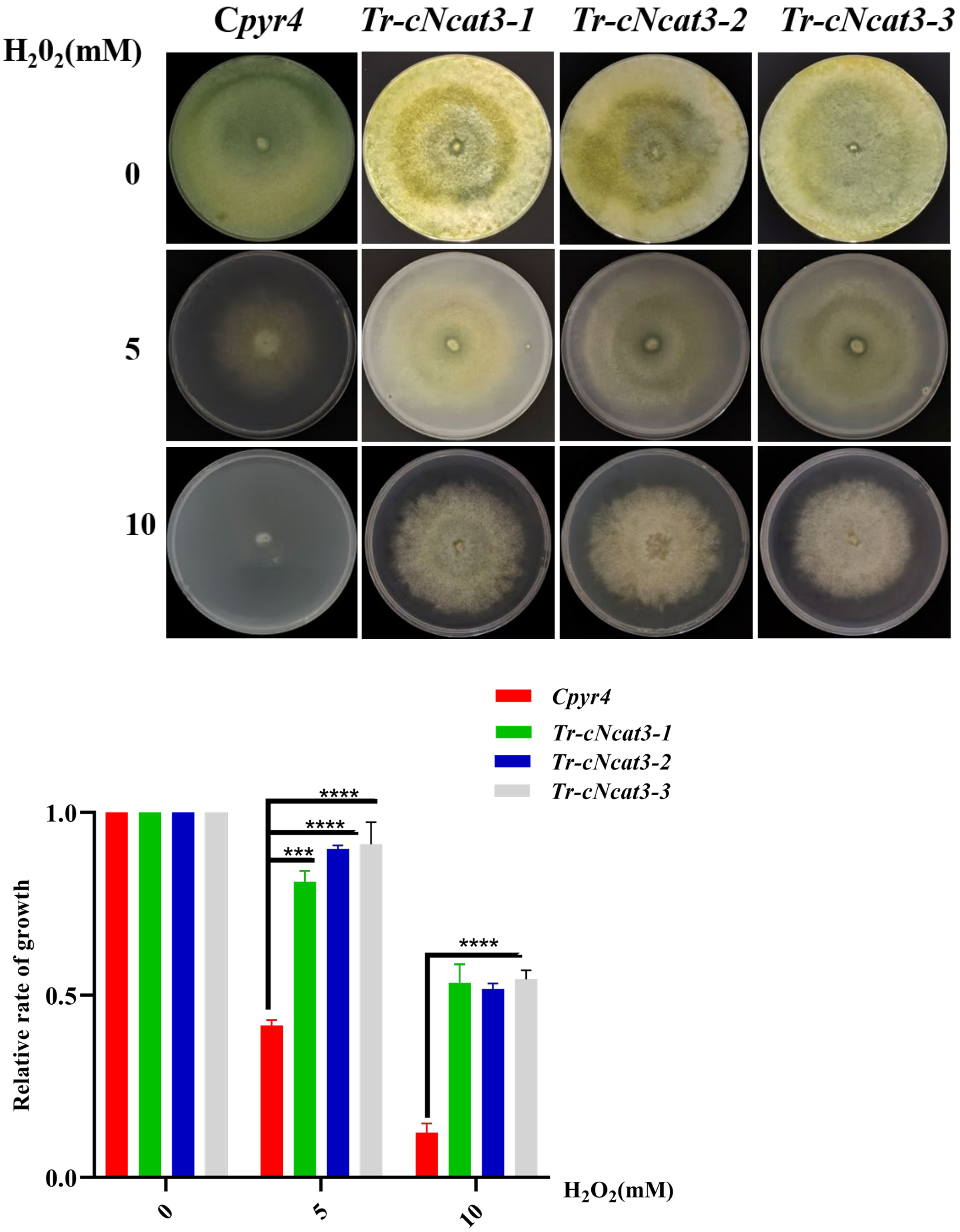


**A**: Mycelial growth of *Cpyr4, Δcel3c*, and *Tr-cNcat3* under 0, 5, 10 mM $H_2O_2$ conditions. A suspension containing 1 × $10^5$ conidia of *T. reesei* was inoculated at the center of a plate and cultured for 72 h under constant conditions of 25 °C. **C**: The relative growth rates of *Cpyr4*, *Tr-cNcat3-1*, *Tr-cNcat3-2* and *Tr-cNcat3-3* under different concentrations of $H_2O_2$. The growth diameter of each strain in 0 mM $H_2O_2$ medium was defined as the reference unit "1". Relative

growth rates were then calculated for strains in 5 mM and 10 mM $H_2O_2$ media. Data represent mean ± SD. Statistical significance was determined by Two-way ANOVA followed by Tukey's multiple comparison test. ns, not significant; *$p< 0.05$; ****$p< 0.0001$.
