# Supplementary material for: Heterologous Expression of a Neurospora crassa Catalase Reprograms the Cellulase System and Enhances β-Glucosidase Production in Trichoderma reesei
Source: J Fungi (Basel). 2026 Jul 25;12(8):554. doi: 10.3390/jof12080554 (PMC13514374; doi:10.3390/jof12080554)
Supplement: Supplementary file 1 [file jof-12-00554-s001.zip › Table S5.pdf]

Supplementary Tables

Table S5 Primers of major transcription factors related to cellulase production.

**Primer name**	**Sequence (5' to 3')**
Actin-F	ACCGCAAGTGCTTCTAAATTCAA
Actin-R	TGCAGAATACACGCGCAATG
cbh1-F	GGTCCAAGTGAGTACGCGAG
cbh1-R	CAAACGGGATGTGAGGGGAT
*cbh*2-F	CAGTTGGATCAGGAACCGCT
*cbh*2-R	ACTGCTTACTTCTGCGGCAT
eg1-F	CCCGTACCGAGTCAACCAAA
eg1-R	GGCAGGGAACTGTGTGATGA
bgl1-F	GAGCAACCCAGATGACCGAA
bgl1-R	AGCACATGACAGAAGCGACA
**V*cat3*-F**	CAACCAGACCGTCGGCCGC
**V*cel3c*-R**	ATTGGTCCTGTTGGAAGCG
**V*3*c-F**	GCTCTCATCCGCGGCAT
**V*3*c-R**	GGTCAATGACGTGGA

*F and R indicate forward and reverse primer, respectively.
